# The Incidence of Molluscum contagiosum among American Indians and Alaska Natives

**DOI:** 10.1371/journal.pone.0005255

**Published:** 2009-04-21

**Authors:** Mary G. Reynolds, Robert C. Holman, Krista L. Yorita Christensen, James E. Cheek, Inger K. Damon

**Affiliations:** 1 Poxvirus and Rabies Branch, Centers for Disease Control and Prevention (CDC), Atlanta, Georgia, United States of America; 2 Division of Viral and Rickettsial Diseases, Centers for Disease Control and Prevention, Atlanta, Georgia, United States of America; 3 Division of Epidemiology and Prevention, Office of Public Health Support, Indian Health Service, USDHHS, Albuquerque, New Mexico, United States of America; The University of Adelaide, Australia

## Abstract

**Background:**

The epidemiology of Molluscum contagiosum (MC) in the United States is largely unknown, despite the fact that the virus is directly communicable and large outbreaks occur. This study provides population-based estimates to describe the epidemiology of MC in the United States among American Indian and Alaska Native (AI/AN) persons. This population was selected because of the comprehensiveness and quality of available data describing utilization of out-patient services.

**Principal Findings:**

Outpatient visits listing MC as a diagnosis in the Indian Health Service National Patient Information Reporting System during 2001–2005 were analyzed to assess patient characteristics, visit frequency and concurrent skin conditions. Outpatient visit rates and incidence rates were calculated based on known population denominators (retrospective cohort). Overall outpatient visit rates were also calculated for the general US population using national data. The average annual rate of MC-associated outpatient visits was 20.15/10,000 AI/AN persons for 2001–2005 (13,711 total visits), which was similar to the rate for the general US population (22.0/10,000 [95% CI: 16.9–27.1]). The incidence of MC-associated visits was 15.34/10,000. AI/AN children 1–4 years old had the highest incidence (77.12), more than twice that for children 5–14 years old (30.79); the incidence for infants (<1 year) was higher than that for adults. AI/AN persons living in the West region had the highest incidence, followed by those in the East and Alaska regions (26.96, 22.88 and 21.38, respectively). There were age-specific associations between MC and concurrent skin conditions (e.g., atopic dermatitis, eczema).

**Conclusions:**

This study highlights the need for periodic population-based measurements to assess trends in incidence and healthcare utilization for MC in the United States. High rates of MC were found among AI/AN persons, especially among children <15 years old. The AI/AN population would benefit from greater availability of effective strategies for prevention and treatment of MCV infection.

## Introduction

Molluscum contagiosum (MC) is a superficial infection of the dermis caused by a virus in the family *Poxviridae*. In otherwise healthy individuals, infection with *Molluscum contagiosum virus* (MCV) results in a benign self-limiting condition marked by the formation of distinctive, persistent dermal lesions that evolve slowly over the course of several weeks to several months. The total time-course of infection may be prolonged due to inadvertent autoinoculation of the virus to other parts of the body. Activities or circumstances that involve skin-to-skin contact (e.g., play, sports such as wrestling, sexual activity, etc.) have been associated with increased risk for infection [1].

Because of the characteristic appearance of MC lesions, diagnosis is generally made without laboratory testing. Often specific treatments or therapies are not pursued for MC infection in immune competent individuals, as lesions will resolve with time, however, mechanical removal (*via* curettage, cryotherapy, or laser treatment) and various topical therapies (including tretinoin, cantharidin, Imiquimod, cidofovir) are sometimes utilized to minimize the duration that lesions are present, particularly on the face or on areas of the body that are subject to heightened irritation. Molluscum contagiosum in persons who have immune compromise—whether due to HIV infection, immunosuppressive drug therapies, or other reasons—can be complicated.

Persons with atopic dermatitis (i.e., eczema) are generally considered to be at greater risk for contracting MC due to immune deficits, compromised skin integrity, or both [2]–[4]. However, investigations into the association between atopic dermatitis and molluscum infection have been observational in nature and have principally addressed the conditions only in children. In general, epidemiologic studies of MC are few and most have focused on rates of infection or risk factors for infection in specific population subgroups. These subgroups have been defined variously by geographic location [5], [6] subject age [7], [8], immunologic status [9], [10] or the type of medical service facility at which they had sought care (e.g., tertiary care facilities, dermatology specialists, etc.) [7], [11]. Provision of population-based incidence rates for MC, which would allow for comparison across studies, is rare.

With this study we seek to provide an extensive description of the epidemiology of MC among American Indian and Alaska Native (AI/AN) persons, a subset of the general US population. The AI/AN population is not anticipated to directly reflect the broader US population with respect to health issues – AI/AN communities are often subject to considerable health disparities—but approximately 57% of the AI/AN population across the United States is served by the Indian Health Service (IHS); therefore utilization of healthcare services through the IHS can provide a broadly encompassing, population-based, view of specific health-related issues including infection with MCV. The IHS National Patient Information Reporting System (NPIRS), which records 100% of clinic and ambulatory care records for all IHS-contracted or operated facilities, captures population-based outpatient visit data through time from across the United States for all AI/AN age groups. This study, which spans five years, represents the largest, most comprehensive molluscum survey performed to date. The MC-associated outpatient visit rate for AI/AN persons is compared with that of the general US population (derived from data available through the National Ambulatory Medical Care Survey [NAMCS] and the National Hospital Ambulatory Medical Care Survey [NHAMCS]). Patient-based MC-associated outpatient visit rates or incidence among AI/AN persons are examined.

## Methods

The IHS direct and contract outpatient visit data for AI/AN persons for calendar years 2001–2005, obtained from the IHS National Patient Information Reporting System (NIPRS), were analyzed [12]. The direct and contract health service outpatient visit data consist of all direct and contract health care outpatient visit records from IHS-and tribally-operated facilities and facilities which are contracted with the IHS or with specific tribes to provide healthcare services to eligible AI/AN persons [12], [13]. All AI/AN persons in this study received IHS-funded health care and may not be representative of all AI/AN persons in the United States.

Molluscum contagiosum-associated outpatient visit records, where MC was listed as one of up to nine diagnoses, were selected for analysis. Visits with MC listed as one of up to three diagnoses were selected from the US ambulatory care data. The *International Classification of Diseases, 9^th^ Revision, Clinical Modification* (ICD-9-CM) code 078.0 was used to define MC [14]. The IHS provided outpatient visits by patient (no unique identifiers were provided to investigators), which enabled the examination of multiple outpatient visits for patients and estimation of the incidence of MC. The patient's first annual MC visit during each year of the study period was used for patient-based analysis to estimate the incidence, reported as number of AI/AN persons with at least one MC-associated outpatient visit per 10,000 AI/AN persons. Because this study only encompasses individuals who have sought healthcare while infected with MCV, it is likely to be a minimum estimate of the true incidence of MC in the AI/AN population. The primary unit of the analysis in this study was the patient, unless otherwise stated.

Both molluscum contagiosum-associated outpatient visits and patient-based outpatient visits for 2001–2005 among AI/AN persons were examined by age group (<1 year [infants],1–4, 5–14, 15–19, 20–44, 45–64 and ≥65 years), IHS region (West Northern Plains [Aberdeen and Billings areas], East Northern Plains [Bemidji area], Southern Plains [Oklahoma area], Southwest [Albuquerque, Navajo, Phoenix and Tucson areas], East [Nashville area], Alaska, and West [California and Portland areas]), sex and etiology. Month of visit and diagnoses listed with a MC diagnosis on the record were examined. Annual outpatient visit rates and incidence rates were calculated and expressed as the number of outpatient visits and patients per 10,000 AI/AN persons, respectively. The IHS user population estimates of AI/AN persons within the IHS healthcare system for fiscal years 2001–2005 were used to calculate the rates [13], [15]. The user population is all AI/AN persons who were eligible to receive IHS-funded healthcare service during the 5-year study period. Rate comparisons among AI/AN persons were evaluated by characteristic using risk ratios (RRs) with 95% confidence intervals (CIs) [16]. Comparisons of the interval in days between outpatient clinical visits by age group were made using the Wilcoxon rank-sum test.

The overall Molluscum contagiosum-associated average annual outpatient visit rates were examined for the general US population. NAMCS and NHAMCS for 2001–2005 were used to examine outpatient visits [17], [18]. The NAMCS and NHAMCS provide data with respect to outpatient visits and provide an assessment of outpatient visits for the general US population. The basic sampling unit for the NAMCS is the physician-encounter. Only visits to the offices of non-federally employed physicians classified by the American Medical Association or the American Osteopathic Association as “office-based, patient care” are included. The NHAMCS comprises a national probability sample of visits to the emergency and outpatient department of non-institutional general and short-stay hospitals. The US national NAMCS and NHAMCS data in this study do not include IHS/tribal facility outpatient visits or identify multiple hospitalizations by patient. For the general US population, the outpatient visit rate was expressed per 10,000 persons using the US Census estimates [19]. Standard errors of NAMCS and NHAMCS estimates were calculated by using SUDAAN software to account for the sampling designs [20], and used to calculate 95% CIs for the rates [16]. If the relative SE of estimates exceeded 0.30 or if the number of unweighted visits in a strata was <30, the estimates were considered unreliable and are not reported [17], [18].

## Results

### Molluscum contagiosum-associated outpatient visits (all visits)

For 2001–2005, the average annual rate of MC-associated outpatient visits for the general US population was estimated to be 22.0/10,000 (95% CI 16.9–27.1) based on an estimated 2,773,792 visits (SE = 396,947). This rate is similar to that for the AI/AN population (20.15/10,000; Table 1), derived from the reported 13,711 MC-associated outpatient visits among AI/AN persons within the IHS healthcare system.

**Table 1:** Characteristics of molluscum contagiosum-associated outpatient visits and average annual outpatient visit rate among American Indians and Alaska Natives, 2001–2005*.

Characteristic	No.	Average annual visit rate/10,000 persons
Total	13711	20.15
Gender†		
Male	6436	19.80
Female	7275	20.47
Age Group (years)		
<1	223	21.85
1–4‡	5731	102.98
5–14	5746	40.41
15–19	570	8.04
20–44	1223	4.91
45–64	174	1.58
≥65	44	1.05
Region§		
Alaska	1736	28.64
East	599	31.08
Northern Plains, East	1115	25.64
Northern Plains, West	2194	23.83
Southern Plains	1769	12.15
Southwest	3433	14.39
West	2865	35.42

* Values reflect the outpatient visits associated with molluscum contagiosum (MC) over the five-year study period (average annual population 1,360,847) among American Indians and Alaska Natives using the IHS healthcare system. A total of 2545 patients had multiple MC-associated visits during this period.

† The average annual rates of MC-associated outpaient visits for males and females in the general US population for 2001–2005 are 20.3/10,000 (95% C.I 13.0–27.6) and 17.8/10,000 (95% CI 10.9–24.7) persons, respectively.

‡ The average annual rate of MC-associated outpatient visits for 1–4 year olds in the general US population is 82.6/10,000 (95% C.I. 44.8–120.4). The low number of visits for other corresponding age groups precludes reliable population-based occurrence estimates.

§ see Figure 2 [map of US with different geographic regions highlighted].

The number and rate of MC-associated outpatient visits in the AI/AN population were similar for males and females, with males experiencing 6436 MC-associated outpatient visits during the 5-year period and females 7275 visits, corresponding to annual visit rates per 10,000 of 19.80 and 20.47, respectively (Table 1). These rates were similar to those for males and females in the general US population (17.8 [95% CI = 10.9–24.7] and 20.3 [95% CI = 13.0–27.6], respectively).

**Figure 1:**
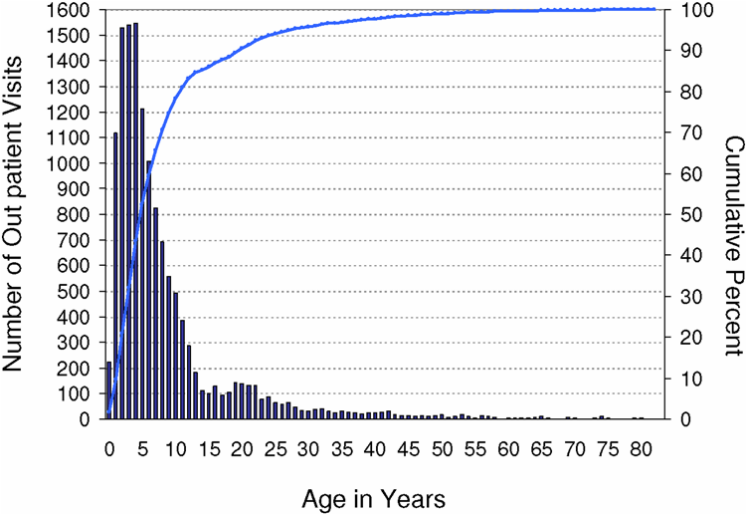
Molluscum contagiosum (MC)-associated outpatient visits among American Indian/Alaska Native persons by age (in years), 2001–2005. The average number of MC-associated IHS/tribal outpatient visits per year over the 5-year period is depicted along the left axis. Cumulative percent is shown along the opposing axis.

Non-infant, pre school-aged AI/AN children (1–4 years of age) exhibited the highest rate of MC-associated outpatient visits (102.98/10,000) compared to the other age groups, though there were nominally more total outpatient visits among school-aged (5–14 year old) children (5731 and 5746 visits, respectively; Figure 1).

**Figure 2:**
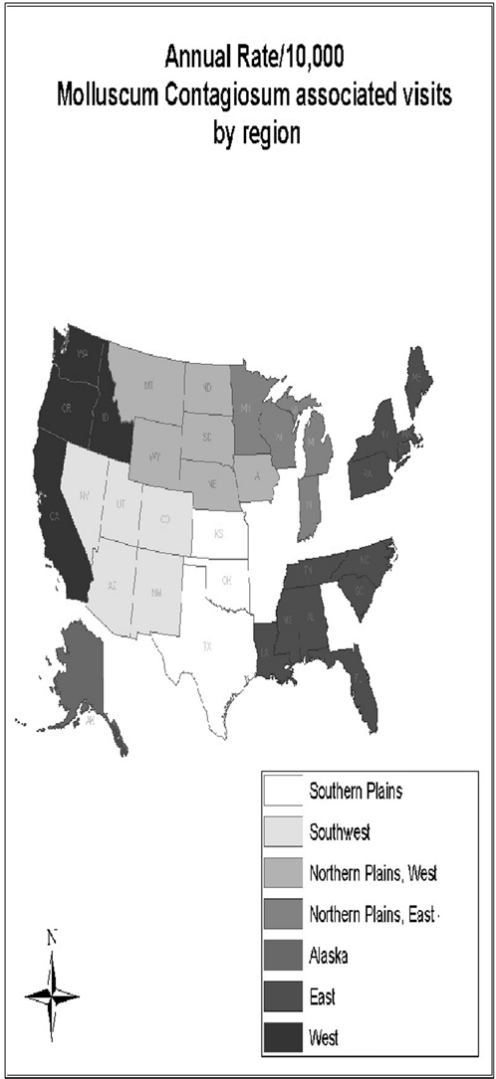
Average annual rate of molluscum contagiosum-associated outpatient visits per 10,000 American Indian/Alaska Native persons by Indian Health Service region, 2001–2005. Southern Plains, 12.1; Southwest, 14.4; Northern Plains, West, 23.8; Northern Plains, East, 25.6; Alaska, 28.6; East, 31.1; West, 35.4. Texas is partitioned into three regions which fall under the jurisdiction of the Southwest, Southern Plains or East IHS regions.

The outpatient visit rates of MC-associated outpatient visits were highest in the West and East regions (35.42 [RR = 2.46. 95% CI 2.34–2.59] and 31.08 [RR = 2.16. 95% CI 1.98–2.36] vs. the Southwest region, respectively), and lowest in the Southern Plains (12.15 [RR = 0.84, 95% CI 0.80–0.89], vs. the Southwest region) (Table 1, Figure 2). The rate of MC-associated outpatient visits was also higher in the Alaska region (28.63) than in the Southwest region (RR = 1.99, 95% CI 1.88–2.1). Visit rates for the West, East and Alaska regions are all higher than the estimated rate for the general US population.

**Figure 3:**
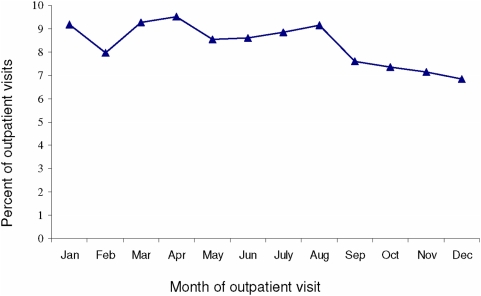
Proportion of molluscum contagiosum-associated outpatient visits occurring January–December among American Indian/Alaska Native persons, 2001–2005.

No annual temporal trends of MC outpatient visits were evident over the five-year period of observation (Figure 3). No statistically significant differences, in terms of region, sex, or age, were observed for outpatient visits between the first two years of the observation period (2001, 2002) and the last (2004, 2005). The average annual MC-associated outpatient visit rate was also not significantly different for 2004–2005 relative to that for 2001–2002 (20.28 and 19.70, respectively; RR = 1.03, 95% CI 0.99–1.07).

### Molluscum contagiosum incidence (molluscum contagiosum-associated visits per patient)

Though typically benign, infection with MCV can result in a protracted condition spanning multiple clinic visits. Therefore, in addition to assessing characteristics related to the total number of MC-associated outpatient visits among AI/AN persons (above), we also assessed characteristics of MC-associated outpatient visits by patient, considering only each patient's first annual MC-associated visit during each year of the study period, termed here mean annual incidence or ‘incidence’; the incidence was also analyzed by age, sex and region (Table 2).

**Table 2:** Characteristics of molluscum contagiosum-associated outpatient visits per patient and average annual incidence among American Indians and Alaska Natives by region, 2001–2005.

Location*																
Characteristic	Alaska		East		Northern Plains, East		Northern Plains, West		Southern Plains		Southwest		West		Overall IHS	
	No.	Rate†	No.	Rate†	No.	Rate†	No.	Rate†	No.	Rate†	No.	Rate†	No.	Rate†	No.	Rate†
**Sex**																
Male	626	21.10	200	21.40	396	18.62	802	17.83	728	10.64	1198	10.62	1025	26.62	4975	15.31
Female	670	21.65	241	24.28	415	18.68	877	18.63	687	8.91	1420	11.29	1156	27.3	5466	15.38
**Age Group (years)**																
<1	27	22.78	14	55.82	6	11.12	36	22.69	29	16.18	51	12.92	35	38.60	198	19.40
1–4	522	97.59	186	123.62	322	96.30	709	85.0	583	51.81	1082	54.24	888	150.08	4292	77.12
5–14	530	40.35	197	50.27	392	43.5	708	35.66	584	20.19	961	18.98	1007	60.24	4379	30.79
15–19	63	9.86	13	7.04	39	8.44	78	7.67	70	4.90	126	5.06	74	8.51	463	6.52
20–44	134	6.36	29	4.07	42	2.65	123	3.68	119	2.22	354	3.99	148	5.05	949	3.81
45–64	17	1.73	1	0.30	10	1.35	22	1.57	25	1.0	35	0.96	26	1.83	136	1.24
≥65	3	0.82	1	0.80	0	–	3	0.64	5	0.47	9	0.65	3	0.59	24	0.57
**Total visits**	1296	21.38	441	22.88	811	18.65	1679	18.24	1415	9.72	2618	10.97	2181	26.96	10,441	15.34

* see Figure 2 [map of US with different geographic regions highlighted].

† Rate of outpatient visits involving molluscum contagiosum per 10,000 AI/AN population within the IHS healthcare system in the specified region (for individuals who had multiple clinic visits involving molluscum contagiosum during the study period, only the first annual visit is included).

During the 5-year period, 10,441 AI/AN persons incurred at least one MC-associated outpatient visit, corresponding to a mean annual incidence of 15.3 cases/10,000 (Table 2). The incidence of MC was similar for males and females regardless of region, except in the Southern Plains area where incidence rates were low and males overall exhibited a significantly higher annual mean incidence than that for females (10.6 and 8.9/10,000, respectively; RR = 1.20, 95% CI = 1.08–1.33). Stratified by age group and region, the highest mean annual incidence of MC was observed for 1–4 year olds in the West and East regions (150.1/10,000 and 123.6/10,000, respectively; RR = 1.21, 95% CI = 1.04–1.42), significantly higher than was observed for the same age group in Alaska, which experienced the next highest rate (97.56/10,000; RR = 1.27, 95% CI 1.07–1.50, Alaska vs East). The highest incidence among infants was recorded in the East and West regions (55.8/10,000 and 38.6/10,000, respectively; RR = 1.45, 95% CI = 0.78–52.69), although the highest rate is based on a relatively small number of observations (14).

Both the number of incident cases and the mean annual incidence of MC demonstrated a trend of modest increase over the 5-year period of observation. The incidence was 19.70 in 2001–2002 and 20.27/10,000 in 2004–2005 (RR = 1.07, 95% CI: 1.03–1.11; Table 3).

**Table 3:** Number of patients with incidence of molluscum contagiosum in the American Indian/Alaska Native population, 2001–2005.*

Year	Patients with at least one MC-associated clinic visit	Change from Previous year	Annual rate/10,000	Change from previous year
2001	1982	na	15.10	na
2002	1925	−3.0%	14.38	−5.0%
2003	2116	+9.0%	15.59	+7.8%
2004	2121	+0.2%	15.33	−1.7%
2005	2297	+7.7%	16.27	+5.8%

* Outpatient visits involving molluscum contagiosum among the AI/AN population within the IHS healthcare system.

### Characteristics of patients with multiple molluscum contagiosum-associated outpatient visits

**Figure 4:**
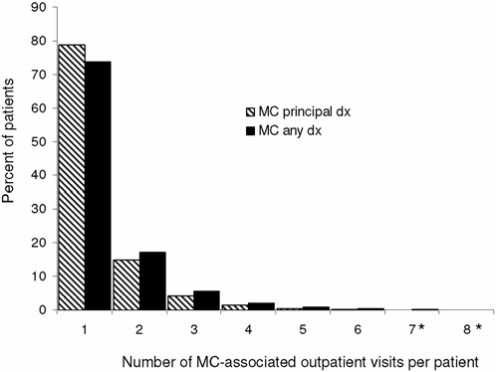
Proportion of patients with molluscum contagiosum (MC) indicated as the principal diagnosis versus ancillary diagnosis, stratified by number of visits per patient among American Indian/Alaska Native persons, 2001–2005. Stripped bars represent proportion of patients with MC as the principal diagnosis; solid bars represent the proportion of patients with MC as an ancillary diagnosis. (*) 0.05 and 0.15% of patients experienced 8 or more MC-associated outpatient visits, respectively.

During the course of the study period, approximately 26% of AI/AN persons (2545) experienced more than one outpatient visit for which MC was indicated as a diagnosis. Overall, the mean number of MC-associated visits per patient in this population was 1.4 (median 1.0), and was similar regardless of whether MC was the principal or ancillary diagnosis for the visit (Figure 4). Two percent of patients in this study had as many as 4 MC-associated outpatient visits during the 5-year study period, and one patient experienced at least 11 visits. Individuals 45–64 years of age were the least likely to have multiple MC-associated outpatient visits; only 16.5% had more than one visit, whereas 28.2% of 1–4 year olds had a diagnosis of MC rendered over the course of multiple outpatient visits (Chi-square p<0.01). The highest mean number of visits per person occurred among the oldest patient in the West and Southwest regions, 4.0 and 3.0, respectively.

Across the population of patients the median number of days between MC-associated visits was lengthy at 42 days (quartiles = 15, 102). The median interval between visits was, however, significantly shorter for those aged 45–64 years, although not different from that for infants (median 24 vs 28.5 days, respectively, p = 0.83). The median interval between visits was longest among patients 1–4 years of age (median 49 vs 42 days for those aged 5–14 years, p = 0.05).

### Molluscum contagiosum with other diagnosed dermatologic conditions

**Figure 5:**
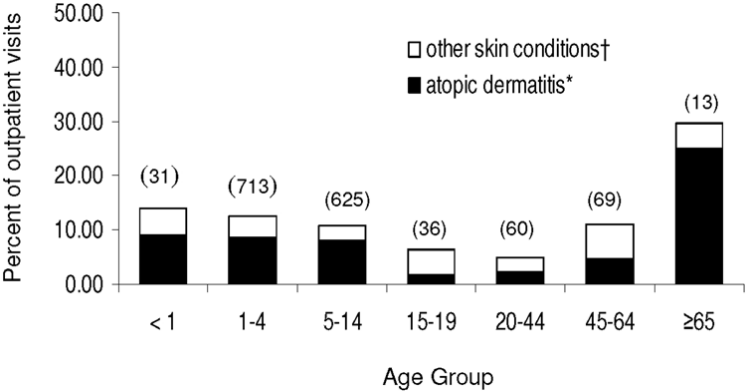
Proportion of molluscum contagiosum-associated outpatient visits among American Indian/Alaska Native persons by age for which concurrent skin conditions were recorded, 2001–2005. Total number of outpatient visits per age groups shown in parentheses. (†) Denotes skin conditions associated with loss of skin integrity (impetigo, diaper rash, unspecificed erythematos conditions, acne); (*) denotes skin conditions potentially associated with immune deficits (eczema/contact dermatitis, atopic dermatitis).

Ancillary diagnoses for all MC-associated outpatient clinic visits among AI/AN persons were assessed to identify significant concurrent skin conditions. Diagnoses associated with atopic dermatitis and conditions indicative of immune deficits with dermatologic manifestations (i.e., eczema) were categorized independently from diagnoses indicating potential loss of skin integrity (i.e., diaper rash, impetigo), and are referred to here as ‘atopic dermatitis’ and ‘other skin conditions’, respectively. The relative frequency of both categories of concurrent skin condition across different age groups is shown in Figure 5. In the AI/AN population, such conditions, particularly ‘atopic dermatitis’, were most prominent among children under 15 years of age and among individuals ≥65 years of age. Conditions associated with loss of skin integrity, such as acne and keratosis, were more prominent among adolescents (15–19 years old) and adults 45–64 years old, but these age groups experienced the lowest overall frequencies of concurrent skin conditions, with only 1.8% of adolescents and 4.6% of adults 45–64 experiencing concurrent conditions, whereas 9.0% and 25.0% of infants and persons ≥65 years, respectively, received such diagnoses.

Patients noted to have atopic dermatitis did not, on average, have a greater number of MC-associated clinic visits.

Ten patients (with 13 visits) had concurrent diagnosis of human immunodeficiency virus (HIV) infection.

### Medical procedures performed in conjunction with Molluscum contagiosum-associated visits

Medical procedures coinciding with MC-associated visits were assessed over the five-year period of observation by reviewing procedure codes recorded for each patient visit (Table 4). During that time, 273 procedures (e.g., vaccine administration, respiratory measure, gynecologic exam, etc.) were recorded in conjunction with MC-associated outpatient visits, but only a single category of dermal procedure was noted. This general category (ICD-9-CM procedure 86.3) encompasses varied forms of mechanical disruption of lesions or skin—cauterization cryosurgery, fulguration and laser excision. (This code excludes biopsy, however). Though rarely performed within any age group, MC-associated outpatient visits were most frequently accompanied by mechanical lesion disruption among patients in the 20–44 and 45–64 year old age groups, accounting for only 4.3% and 9.2% of visits, respectively.

**Table 4:** Number of molluscum contagiosum-associated outpatient visits for which a mechanical removal procedure was performed in the American Indian/Alaska Native population, 2001–2005.*

Age (years)	Number (%) of visits involving procedure †
<1	0 (0)
1–4	66 (1.15)
5–14	122 (2.12)
15–19	16 (2.18)
20–44	52 (4.25)
45–64	16 (9.20)
≥65	1 (2.27)
Total	273

* Outpatient visits involving molluscum contagiosum among the AI/AN population within the IHS healthcare system.

† Included under this procedure category are cauterization cryosurgery, fulguration and laser excision.

There we no prescribed medications for MC treatment noted.

## Discussion

Despite the ubiquity of MCV throughout the world, few recent studies have addressed incidence trends or burden of infection associated with this virus, and none that we know of have provided population-based rates to describe incidence or health care utilization. The purpose of this study has been to provide a comprehensive view of epidemiology of MC across different age groups and in different parts of the United States, by utilizing the health service information for AI/AN persons available through the IHS, which affords a more detailed accounting of outpatient service utilization than is available for the general US population.

Our findings point to a relatively high overall MC-associated outpatient visit rate in the AI/AN population (20.15/10,000 AI/AN persons). This rate is comparable to that estimated for the US population overall, although the rate for AI/AN persons living in the West, East and Alaska regions may be higher than that for the general US population.

A national survey of MC-associated physician visits performed over two decades ago, using data obtained from the National Disease Therapeutic Index Survey (NDTIS) of the United States demonstrated an increase in the proportion of MC-associated clinic visits (per total clinic visits) during a 18-year period from 1966 to 1983, ranging from 1.2 to 11 MC-associated visits per 100,000 clinic visits [21]. In contrast, here, we saw relatively high but stable incidence rates over the 5-year period of observation. It is important to note, however, that ‘clinic visits’ were used as the denominator for MC-associated health care utilization rates in the NDTIS study, whereas in this study we report annual MC-associated outpatient incidence per 10,000 AI/AN persons, therefore rates are not directly comparable.

In addition to high overall incidence, we note relatively high incidence among specific population sub-groups, notably 1–4 year olds in the East and West regions where incidence rates of 123.62/10,000 and 150.08/10,000, respectively, were reported. These incidence rates are comparable to that for chickenpox prior to initiation of vaccination (1980–1990), which was estimated to be 149.8/10,000 [22]. Recent studies which measured age-related health care utilization for MC concluded that rates of health care utilization, and overall numbers of MC-associated visits were highest among children 2–5 years old [7], [23]. Formerly, onset of schooling was typically viewed as a social risk factor for acquisition of MC, particularly in developed, affluent nations [1]; a study performed in Holland in the late 1980s provided evidence consistent with this idea [11]. However, increased utilization of child day care in the United States during the last two decades has generally lowered the age of socialization (outside the immediate household) among children across most socio-economic strata [24]. It bears further consideration whether the observed elevated burden of infection observed among pre school-aged children in this study reflects general tendencies toward communal care of very young children in the AI/AN population, or possibly heightened utilization of child daycare.

Independent of age, both poverty and crowded living quarters have been hypothesized as general risk factors to account for perceived higher rates of MCV particularly in the tropics [1], [8]. Moisture and humidity have been implicated as relevant climatic factors as well [1], [5], [25], but the strength of association between poverty, geography or climate, and incidence of infection with MCV remains undetermined. In our study, overall incidence of MC-associated clinic visits was observed to be highest in the West, East and Alaska regions, and lowest in the Southwest and Southern Plains regions, the latter of which are arguably the areas with warmest climate. These regional differences in incidence were stable over the 5-year period of observation; it seems unlikely that the observed differences could be attributable to climate alone.

For individual AI/AN patients, however, the presence of concurrent skin conditions was often attendant MC-associated visits. For purposes of this study, these conditions were broadly categorized into those associated with immune deficits with dermatologic manifestations, for example atopic dermatitis and eczema, versus those suggestive of breaches in skin integrity, such as acne and impetigo. Conditions in either category could predispose persons to MCV infection (eczema is a risk factor for severe complications from infection with poxviruses and other cutaneous viral pathogens) but, notably, the relative frequencies of each category of skin condition varied in line with MC patient age. Atopic dermatoses were diagnosed concurrently with MC in approximately 8% of all persons under the age of 14, and in ∼18% of persons ≥65 years of age, but these diagnoses were relatively infrequent among persons 15–64 years old. Conversely, among 15–64 year olds, a greater proportion of patients were diagnosed with acne or erythematos conditions, both associated with loss of skin integrity. These observations suggest that risk reduction messages tailored for AI/AN patients of specific ages could be useful in preventing MCV infections. (For example, teenagers with acne should be enjoined to take special care when interacting with a younger sibling that has MCV infection.)

This study demonstrates that within the AI/AN population in the United States there is appreciable utilization of health care services for MCV infection, but there appears to be a relative absence of use of specific medical interventions which could be employed to diminish the consequences of infection (although prescription treatments were not specifically assessed). Pediatric and adult treatment options include curettage and application of various topical agents [26], [27]. The risk of MCV transmission and subsequent infection (either to oneself or to others) can be lessened by successful use of interventions that hasten the resolution of lesions. But physicians need always to balance the potential value of the intervention against the potential risks, i.e., scarring, pain, etc. [26].

The IHS/tribal and the general US population outpatient visit data are very useful for evaluation, although there are some limitations to the data. Only AI/AN persons who had received medical evaluation for MC are included in the estimate in this study, therefore the incidence rate reported here may be an underestimate of the true incidence rate of MC occurrence in the AI/AN population, and regional variations in health-care seeking behavior may exist. Also, diagnoses may be incomplete, miscoded or vary by region. The AI/AN user population is an estimate of the number of AI/AN persons who are eligible to utilize the IHS healthcare system and may not include all AI/AN persons who seek medical consultation. Further, the AI/AN persons in this study are not representative of all AI/AN persons in the United States. For the general US population, only the overall MC outpatient visit rates were estimated due to the small number of MC visits in the surveys. Also, multiple outpatient visits for individual patients are not identified; therefore, the incidence of MC can not be estimated for the general US population.

In sum, this study highlights the need for periodic population-based measurements to assess long-term trends in incidence and healthcare utilization for MC, particularly as regards the age of initial MCV acquisition, associations with other concurrent skin conditions, and availability and use of medical interventions. Though this study specifically addresses MC-associated health care utilization in the AI/AN population in the United States, our results highlight features of MC epidemiology that merit further consideration and evaluation for other populations in the United States and elsewhere. Because the need seems great, the AI/AN population should benefit from community outreach regarding MC prevention, particularly in very young children.
